# Stroke promotes the development of brain atrophy and delayed cell death in hypertensive rats

**DOI:** 10.1038/s41598-020-75450-6

**Published:** 2020-11-19

**Authors:** Mohammed A. Sayed, Wael Eldahshan, Mahmoud Abdelbary, Bindu Pillai, Waleed Althomali, Maribeth H. Johnson, Ali S. Arbab, Adviye Ergul, Susan C. Fagan

**Affiliations:** 1 Clinical and Experimental Therapeutics, College of Pharmacy, University of Georgia, 914 New Baillie Street, HM Building Room 116, Augusta, GA 30901 USA; 2 Charlie Norwood VA Medical Center, Augusta, GA USA; 3 Department of Physiology, Medical College of Georgia, Augusta, GA USA; 4 Department of Neuroscience and Regenerative Medicine, Augusta, GA USA; 5 Georgia Cancer Center, Augusta, GA USA; 6 Department of Pathology and Laboratory Medicine, Medical University of South Carolina, Charleston, SC USA; 7 Ralph H. Johnson VA Medical Center, Charleston, SC USA

**Keywords:** Neurology, Diseases of the nervous system, Neurodegeneration, Stroke

## Abstract

Post-stroke cognitive impairment (PSCI) is a major source of disability, affecting up to two thirds of stroke survivors with no available therapeutic options. The condition remains understudied in preclinical models due to its delayed presentation. Although hypertension is a leading risk factor for dementia, how ischemic stroke contributes to this neurodegenerative condition is unknown. In this study, we used a model of hypertension to study the development of PSCI and its mechanisms. Spontaneously hypertensive rats (SHR) were compared to normotensive rats and were subjected to 1-h middle cerebral artery occlusion or sham surgery. Novel object recognition, passive avoidance test and Morris water maze were used to assess cognition. In addition, brain magnetic resonance images were obtained 12-weeks post-stroke and tissue was collected for immunohistochemistry and protein quantification. Stroked animals developed impairment in long-term memory at 4-weeks post-stroke despite recovery from motor deficits, with hypertensive animals showing some symptoms of anhedonia. Stroked SHRs displayed grey matter atrophy and had a two-fold increase in apoptosis in the ischemic borderzone and increased markers of inflammatory cell death and DNA damage at 12 weeks post-stroke. This indicates that preexisting hypertension exacerbates the development of secondary neurodegeneration after stroke beyond its acute effects on neurovascular injury.

## Introduction

Stroke has recently become the fifth leading cause of death in the United States^1^. While stroke mortality has been steadily declining over the last decade due to the continuous improvement in health care standards^2^, the number of stroke survivors with residual disability is steadily increasing^3^. Ischemic stroke is a condition characterized by an initial ischemic event that deprives brain tissue from blood supply and oxygenation, that is sometimes followed by reperfusion, leading to irreversible brain damage and subsequent motor and cognitive impairment^4^.

Post-stroke cognitive impairment (PSCI) is a condition that affects up to two-thirds of patients following ischemic stroke, with up to one third eventually developing dementia^5,6^. Although PSCI is highly prevalent among stroke survivors, there is evidence suggesting that the criteria for diagnosis may underestimate the frequency of dementia and cognitive decline among stroke survivors^7,8^. While it was traditionally thought that the cognitive impairment results from the recurrence of ischemic insults, newer evidence suggests that a very substantial portion of this impairment results from neuronal pathogenesis^9^. Published data from a large, NIH-funded, epidemiologic trial showed that patients, in addition to acute changes, suffer from a slowly progressive cognitive decline after a single-stroke lesion^10,11^. This continuous deterioration occurs even in the absence of any evidence of new ischemic injuries^12,13^.

Diagnosis and characterization of clinically apparent PSCI has proven to be a challenging task, owing to the heterogeneity of the condition itself. The incidence of PSCI and its severity depends largely upon the morphology of the vascular injury (focal or multifocal; large or small vessel), volume of brain tissue affected by ischemia and, most importantly, the location and number of lesions^14^. Histopathological studies have shown a clear link between the vascular aspects of brain injuries affecting cognition and the neurodegenerative aspects that resemble Alzheimer’s disease pathology^15^.

Hypertension is the most commonly occurring modifiable risk-factor for stroke worldwide and is being increasingly recognized as a risk factor for the development of PSCI^15,16^. Chronic hypertension, particularly midlife high blood pressure (BP), has been associated with an increased risk for cognitive decline, vascular dementia and Alzheimer’s disease^17^. One of the mechanisms by which hypertension is believed to contribute to the development of cognitive impairment is exposing the cerebral microvasculature to pulsatile pressure causing tearing of the brain vascular endothelium and smooth muscle cells leading to lipohyalinosis and fibrinoid necrosis^18^. Clinical studies on hypertensive patients have shown that acute disruption of blood perfusion can lead to the formation of lacunar infarcts, while chronic ischemia can lead to the development of white matter lesions which are associated with the development of cognitive impairment^19,20^.

In this study, we aimed to determine the role of hypertension in the development of cognitive impairment following experimental stroke in rats by comparing normotensive (Wistar) rats to spontaneously hypertensive rats (SHR). We hypothesized that hypertension plays a key role in exacerbating PSCI by inducing neurodegenerative processes, causing SHRs to be more susceptible to PSCI compared to normotensive animals.

## Methods

**Figure 1:**
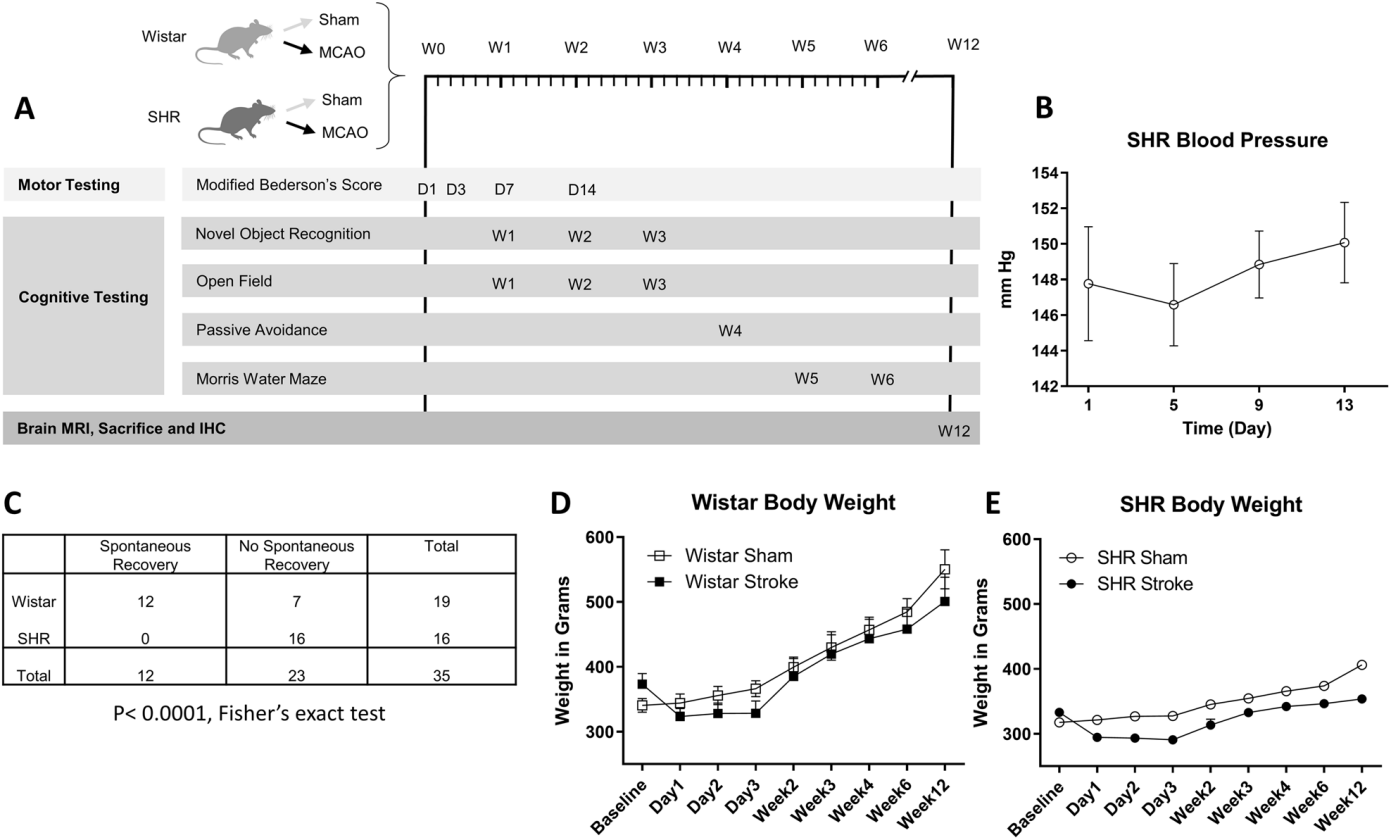
Hypertension increases stroke mortality and reduces spontaneous recovery. (**A**) Experimental design showing different time points for behavioral testing. (**B**) The mean arterial blood pressure of a cohort of SHR animals of the same age and weight of our stroke animals was followed for 2 weeks to establish their blood pressure (n = 6). (**C**) 63% of the Wistar animals achieved complete spontaneous post-stroke recovery after 24 h of MCAO, compared to 0% for SHR animals. (P < 0.0001, Fischer’s exact test) (**D**) & (**E**) Body weight was recorded for 12 weeks following MCAO comparing sham animals from both strains to stroke animals (n = 6–12 per group).

The data that support the findings of this study are available from the corresponding author on reasonable request. All animal experiments were approved by the Institutional Animal Care and Use Committee of the Charlie Norwood VA Medical Center, Augusta, Georgia. All methods were performed in accordance with the relevant guidelines and regulations. Overall experimental design can be found in (Fig. 1A).

### Data analysis

Statistical analyses were performed using SAS version 9.4 (SAS Institute, Inc, Cary, NC) by a biostatistician (Johnson MH). All data are presented as means ± SEM. Unless otherwise mentioned, all results presented were analyzed by 2-way ANOVA (2 Strain (Wistar vs. SHR) X 2 Surgery (sham vs. stroke)), with p-values for the tests presented as tables on the graphs. Letters in the graphs represent the results of Tukey post-hoc multiple comparisons tests for significant ANOVA results where pairs of means with different letters are significantly different. Otherwise, comparisons of two groups were analyzed by student t-test for parametric values, and Wilcoxon’s signed rank test for non-parametric values. Results are considered significant at a Type I error rate of 5%.

### Experimental animals

Both male Wistar and SHR animals were purchased from Charles River (Wilmington, MA) and were housed (1 rat per cage) in pathogen free, temperature-controlled facility (24 ± 1 C; 12–12-h light–dark cycle) with access to standard chow and water ad libitum.

### Blood pressure monitoring

SHR animals are known to develop hypertension in a progressive fashion. To prove that our SHR animals have developed an elevated mean arterial blood pressure, we used an age matched cohort of 6 male SHR animals implanted with telemetry devices, measuring their mean arterial blood pressure, using continuous BP telemetry with transmitters (Data Sciences International, St Paul, MN, USA). Description of this method is adapted from our previously published work Ahmed et al.^21^. The transmitters were implanted in rats, under 3–5% isoflurane inhalation anesthesia, as reported previously according to the manufacturer’s specifications^22^. In brief, a midline incision was performed to expose the abdominal aorta, which was shortly occluded to allow insertion of the transmitter catheter, which was secured in place using tissue glue. The incision was closed using non-absorbable suture (3–0). Rats were returned to their individual cages and allowed to recover from surgery for 10 days. By placing cages on top of the telemetry receivers, arterial pressure waveforms were continuously recorded throughout a 14-day period.

### Transient middle cerebral artery occlusion (tMCAO) surgery

Male Wistar and SHR rats (age: 10–14 weeks) (body weight: 320–400 g) were subjected to 60-min of tMCAO using 4–0 silicon coated nylon suture (Doccol 403756)) as previously described^22^. The original source of method description is our previously published work, Eldahshan et al.^23^ . Briefly, the animals were anesthetized using 2–5% isoflurane, a ventral mid-line neck incision was made, the right common carotid artery (CCA) was exposed, and the external carotid artery (ECA) was ligated and cut. The suture was advanced from a small nick at the ECA into the internal carotid artery (ICA) until a mild resistance was encountered, indicating the branching of the anterior and middle cerebral artery. The suture was tied in place for the duration of the occlusion and the animals were allowed to recover from anesthesia. Several minutes before the end of the occlusion time, the animals were re-anesthetized, the suture was removed for reperfusion and the small nick at the ECA was permanently ligated. In sham surgeries, the CCA was isolated and manipulated without cutting or insertion of the suture and the skin was closed.

We defined a failed MCAO surgery as: “Insertion of the suture that did not succeed in producing an ischemic damage as evident from the lack of motor deficits after reperfusion, possibly due to an incomplete occlusion of the origin of the MCA”.

### Randomization and blinding

Animals were randomized to either the sham or stroke group using a stratified block randomization method, in which animals were arranged into strata based on body weight, with each stratum being divided equally between groups, to achieve an equal distribution of body weight (sham vs. stroke) within strain, before surgery. All behavioral, histochemical and molecular assessments and analyses were performed by blinded investigators. Animals were assigned numbers 1–50 after randomization, with their strain and surgery unknown to all investigators except the surgeon. Animals were assigned to groups A-D based on strain and surgery, and group assignments were revealed to the investigators after the conclusion of the tests and analyses.

### Inclusion and exclusion

To ensure the success of the surgery, animals were tested for unilateral paresis immediately following reperfusion to exclude any animals that did not show a significant deficit. No animals were excluded for failed MCAO. Animals that showed spontaneous recovery at 24 h post-surgery were excluded. Our inclusion criteria were: (1) At least 5% loss in body weight at 24 h after stroke and (2) a score of 6 out of 8 or less on a 4-task neurological score based on the Bederson test. Animals that failed to satisfy one of these criteria at 24 h post-stroke were deemed spontaneously recovered.

## Assessment of functional outcome

### Body weight

As we described in our previously published work Ahmed et al.^21^, weight monitoring is an extremely important tool that serves as an independent and unambiguous measure of an animal’s overall health and welfare, specifically after stroke. For our studies, animals were weighed before surgery and then daily after stroke for the first 14 days, then once a week until the day of sacrifice. All animals selected for the study were in the range of 300–400 g body weight at baseline.

### Neurobehavioral testing

All neurobehavioral tests were conducted, recorded, and analyzed in a blinded manner.

### Sensorimotor testing

To assess sensorimotor function, animals underwent an 8-point modified neurological assessment modified from the Bederson protocol^23^ at days 1, 3, 5, 7 and 14 post surgery. Furthermore, motor recovery was assessed via measuring the locomotor activity during the open field and Morris water maze (MWM) tests.

### Modified Bederson score

Animals were assessed neurologically on an 8-point scale measuring 4 basic functions (spontaneous rotation, resistance to lateral push and fore and back paw flexion) with a score of 2 points awarded in each category for an animal exhibiting a natural response, a score of 1 point for mildly impaired animals and a score of 0 for strongly impaired animals. Higher scores indicate better performance with a maximum possible score of 8/8 and a minimum possible score of 0/8.

### Cognitive testing

Cognitive tests were performed according to the design in (Fig. 1). Special consideration was taken for cognitive tests to allow sufficient period of time to prevent different tests from affecting one another. Open Field and Novel Object Recognition (NOR) were allowed to be performed on the same days as these tests were of minimal invasiveness, measuring the spontaneous activity of the animals. However, invasive tests such as passive avoidance (PAT) and exhausting tests such as the MWM were done individually. The NOR was performed to evaluate non-spatial working memory^24–27^, while the passive avoidance test (PAT) assessed associative learning and reference memory^28,29^.

### The novel object recognition (NOR) test

The original source of method description is our previously published work, Ahmed et al.^21^. The NOR test was performed to evaluate non-spatial working memory related to frontal-subcortical circuits. This test was based on the spontaneous tendency of animals to interact with a novel object more than a familiar one and consisted of 2 trials separated by a retention period. On the designated test day, animals were first subjected to an acquisition/sample trial, where the animal is presented with 2 identical (sample) objects and allowed to explore for 15 min. Following sample object exposure, the animal was returned to its home cage for a 1-h retention period. The 2nd preference trial/test session (5 min), which follows the retention period, was conducted in the same manner as the 1st trial, except that a new/novel object replaces one of the familiar/sample objects. The arena and objects were cleaned after each session with 70% ethanol. The time spent in exploring each object during the preference trial/test session was recorded and the discrimination index, which is the difference in exploration time for the objects divided by total time of exploration, was calculated. The discrimination index (DI) and the recognition index (RI), which is the time spent exploring the novel object relative to the total time of exploration, were taken as indicators of working memory.

$$ {\text{Discrimination }}\,{\text{index}}\,\left( {{\text{DI}}} \right) = \left( {{\text{TN}} - {\text{TF}}} \right)/\left( {{\text{TN}} + {\text{TF}}} \right) $$

$$ {\text{Recognition}}\,{\text{ index}}\,\left( {{\text{RI}}} \right) = {\text{TN}}/\left( {{\text{TN}} + {\text{TF}}} \right) $$

▪ Time spent interacting with the familiar object (TF).

▪ Time spent interacting with the novel object (TN).

The required exploratory criteria was that animals should spend between 20–80% of the time exploring the objects out of the 5 min. Objects used were chosen as previously described^30^. Briefly, objects used were unified between animals and chosen according to recommendations of Heyser and Chemero in that they were symmetrical and transparent, and made of odorless, durable, and easy to clean plastic and glass.

### The passive avoidance test (PAT)

The original source of method description is our previously published work, Ahmed et al.^22^. The passive avoidance test was used to assess aversive associative learning and related reference memory. For this test, one of the compartments of a Y-maze was equipped with a metal floor connected to an electric circuit box, adjusted to deliver brief, moderate intensity electric shocks (3 s duration, 0.75 mA). For the acquisition trial, the shock compartment/arm was blocked, and the animal placed in one of the “safe” arms and allowed 10 min to explore the 2 open arms. Upon completion of 10 min, the door blocking the shock arm was opened allowing the animal to enter. Once the animal had fully entered the shock arm, its initial latency was recorded, and it received a brief electric shock before being returned to its cage. After a 72-h retention period, the test trial was conducted. This was performed in a manner similar to that of the acquisition trial except that the foot shock was omitted, and all 3 arms were accessible to the animal from the start. The difference, between training and test sessions, the latency to enter the shock arm was used as a measure of retention. This latency was recorded for up to 300 s, as the index of long-term aversive associative memory consolidation.

### The Morris water maze (MWM)

The MWM test was used to assess spatial learning and long-term memory. The original source of method description is our previously published work, Ahmed et al.^24^. All water maze tests were conducted in a large circular pool of water, 120 cm in diameter, 55 cm height, filled to a depth of 35 ± 1 cm with water at 25 ± 2 °C. This was separated into quadrants designated northeast (NE), northwest (NW), southeast (SE) and south-west (SW), based on the 4 equally spaced cardinal points N (North), S (South), E (East), and W (West) around the edge of the pool. One of these quadrants contained a transparent escape platform (10.5 cm diameter), submerged 1.5 cm below the water surface and obscured from view. Visual extra-maze cues were mounted to aid spatial navigation.

### MWM training/learning sessions

The initial training consisted of a single daily session of 8 trials (60 s each) for the first day, followed by a daily session of 4 trials per day for 3 consecutive days, for a total of 20 training trials. Each trial consisted of releasing the rat into the water from 1 of the 4 starting locations and allowing it to find the platform. If they did not reach the platform within 60 s, they were gently guided to it and kept there for 10 s, then removed. Trials were spaced at least 1 min apart. All trials were recorded, and video tracked by the computerized tracking system Etho-Vision XT 7 (Noldus, Leesburg, VA, USA). This automated system monitored animals’ swim patterns and calculated mean escape latency (s), total distance travelled to target (cm), and velocity to target (cm/s). Data from all training sessions were pooled for each individual animal, evaluated and compared between groups at the different time points.

### MWM spatial reference memory test

Spatial reference memory was assessed with a probe test 24 h after the last daily session. For this test, all procedures were kept the same as during training, except that the platform was removed, and rats were allowed to swim for 60 s in an attempt to find it. Performance was evaluated by measuring time spent in the target quadrant/zones, proximity to the target location, and initial latency to the target zone. The target zone was centered on the platform location and was 3 times bigger.

### Magnetic resonance imaging (MRI)

Method description is adapted, with changes, from our previously published work, Ahmed et al.^25^. To determine the changes in the brain, ventricular volume, and the presence of white matter hyperintensities, animals underwent T2 -weighted and fluid attenuated inversion recovery (FLAIR), 8 weeks after MCAO. This was performed using a horizontal 7.0 T BioSpec MRI spectrometer (Bruker Instruments, Billerica, MA) equipped with an 8.9-cm micro imaging gradient insert (100 G/cm. All T2-weighted MRI and FLAIR images were obtained at Augusta University by the Core Imaging Facility for Small Animals (CIFSA). All MRI images were registered DICOM sequences, analyzed using FIJI. Total brain volume as well as hemispheric and ventricular volumes (regions of interest), were determined on binarized sequences obtained by thresholding. For each region of interest, the volumes were calculated by adding the areas measured on each slice (11 slices total) and multiplying it by the slice thickness (1 mm in all cases).

### Animal sacrifice and tissue collection

The original source of method description is our previously published work, Ahmed et al.^21^. At week 12, animals were anesthetized with IP ketamine/xylazine and transcardially perfused with 300 ml of PBS. Animals were decapitated, and their brains collected. Brains were sliced into 2 mm coronal sections, using a glass slicer matrix (Braintree Scientific, Braintree, MA, USA) and sections were labeled from A to F, anterior to posterior. Sections A and B, from brain matrix, were snap frozen and kept for molecular testing. The remaining brain tissue was immersed in 10% formalin (Fischer Scientific, Waltham, MA, USA) for 48 h and then transferred to a 30% sucrose solution until taken for frozen sectioning.

### Immunohistochemistry (IHC)

Frozen brain sections (5 μm thick) were processed and stained following a standard technique. Method description is adapted from our previously published work, Jackson et al.^26^. Briefly, sections were washed in PBS plus 0.03%/Triton X-100 with gentle agitation, blocked for 2 h in 10% normal goat serum/1% BSA, and incubated overnight at 4 °C with anti-IBA-1 (Ionized calcium-binding adaptor molecule 1, 1:500, Wako, Japan) and anti-GFAP (Glial fibrillary acidic protein, 1:500, Sigma-Aldrich, Burlington, MA). After washing, slides were incubated with their appropriate fluorophore-conjugated (Texas Red, Alexa 488) secondary antibodies (1:1000; Abcam) for 1 h at room temperature and washed and cover-slipped with Fluoroshield mounting medium with DAPI (Millipore-Sigma, Burlington, MA). Imaging was performed using the Keyence Microscope (Itasca, IL). 20× magnification images were obtained from the ischemic border zone (penumbra) of the stroked animals and compared to the cortex of the sham animals. Images were analyzed by using the ImageJ software (NIH).

## Results

### Hypertension increases stroke mortality and worsens stroke outcome

As SHRs are known to develop hypertension over the course of the first few months of their life, it was important to make sure that our animals had already developed hypertension at the time of stroke (10–12 weeks of age). For this purpose, we assessed the mean arterial blood pressure of a cohort of 6 male SHR animals over the course of 2 weeks, via telemetry. All of the animals developed elevated mean arterial blood pressure with an average ranging between 147–150 mm Hg compared to a normal expected value of 96.5 ± 10.7 for Wistar rats under normothermia^27^ (Fig. 1B).

Next, we evaluated the impact of ischemic stroke. Based on preset criteria (see “Methods”), 12 out of 19 animals from the Wistar group spontaneously recovered at 24 h while there was no spontaneous recovery in the SHR group (P < 0.0001, Fisher’s exact test) (Fig. 1C).

Animals were weighed regularly over the course of the study to assess their recovery after the surgery. Animals from both strains started regaining weight 3 days after stroke, with Wistar animals continuing to gain weight until the end of the study, while SHR animals plateaued at an average of less than 400 g (Fig. 1D,E). Three animals from the Stroke SHR group were sacrificed prior to the completion of the study due to excessive weight loss (> 30%), in accordance with the study protocol. Survival curves are displayed in (Supp. Fig. 1) (P = 0.03, Log-rank test for trend).

**Figure 2:**
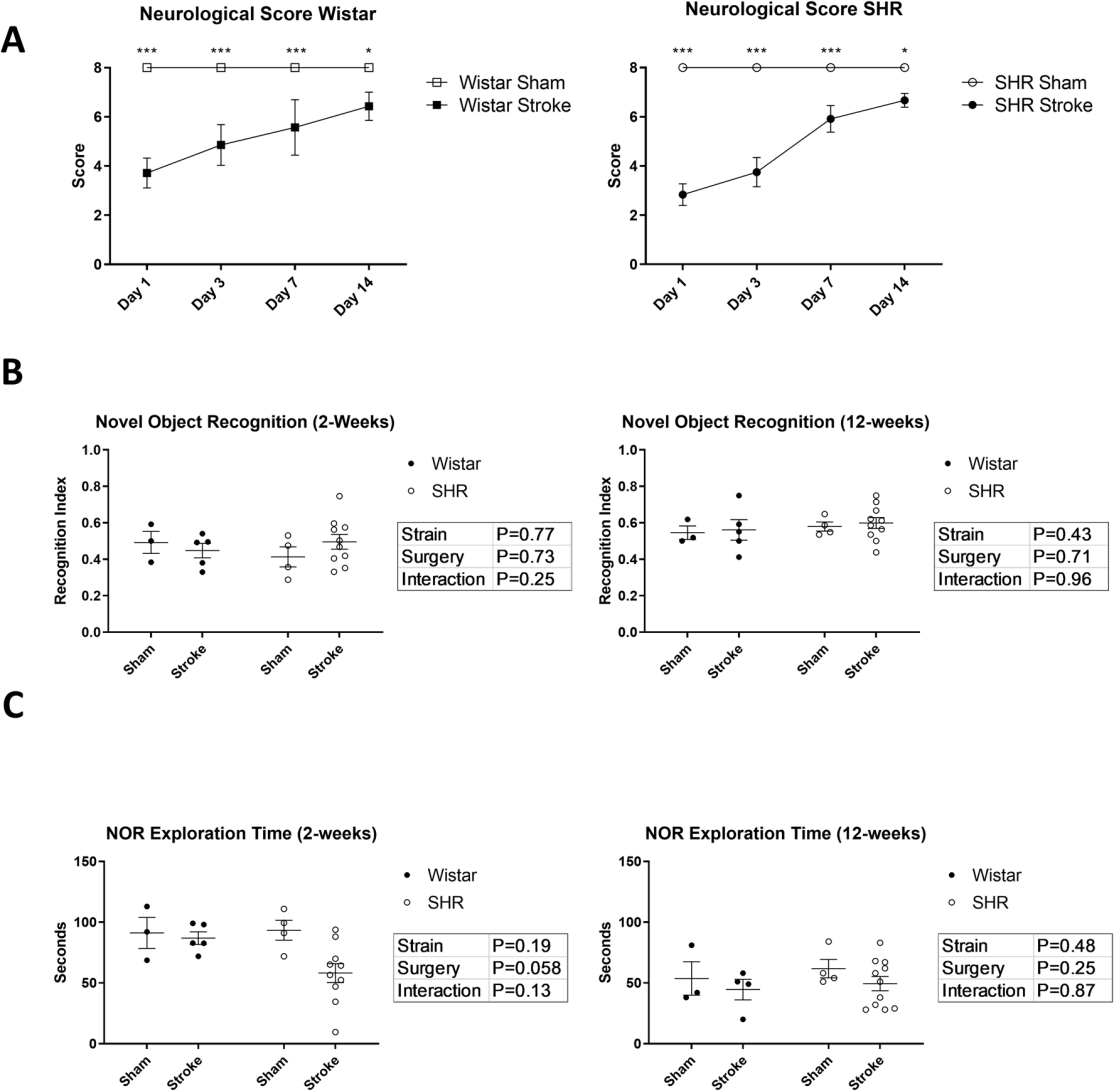
Hypertension causes slower sensorimotor recovery. (**A**) Both strains had significant neurological impairment (Modified Bederson’s Score) starting 24 h after stroke and up to day 14. (n = 6–12 per group) (One sample t-test compared to the maximum score of 8). (**B**) There was no significant difference between the 4 groups in object discrimination in the novel object recognition test at 2- and 12-weeks post MCAO. (n = 6–12 per group). (**C**) SHRs showed a trend for reduced total object exploration time at 2-weeks post stroke (p = 0.058, 2-way ANOVA). The difference disappeared at 12-weeks post-stroke. (n = 6–12 per group).

To assess motor recovery, animals were assessed neurologically, in a blinded manner, on a modified Bederson score, as described in the methods. Based on this assessment, both strains of animals recovered to near their baseline during the course of the first 14 days of follow-up (Fig. 2A). All animals were able to ambulate freely. Animals were assessed at the end of week 1 post-stroke using the open field test, without significant differences from sham (Supp. Fig. 2).

### Hypertension does not affect working memory in the novel object recognition test

Short-term memory was assessed using the novel object recognition test (NOR). Animals that spent less than 20% of the total time exploring the objects, had their trials excluded from the results (0 trials). Neither Wistar nor SHR animals showed a significant impairment in recognizing the novel object at weeks 2 or 12 post-stroke (Fig. 2B). Total exploration time was reduced in SHR animals (Fig. 2C) but it did not achieve significance.

### Hypertension induced depressive symptoms without affecting memory and learning in Morris water maze

**Figure 3:**
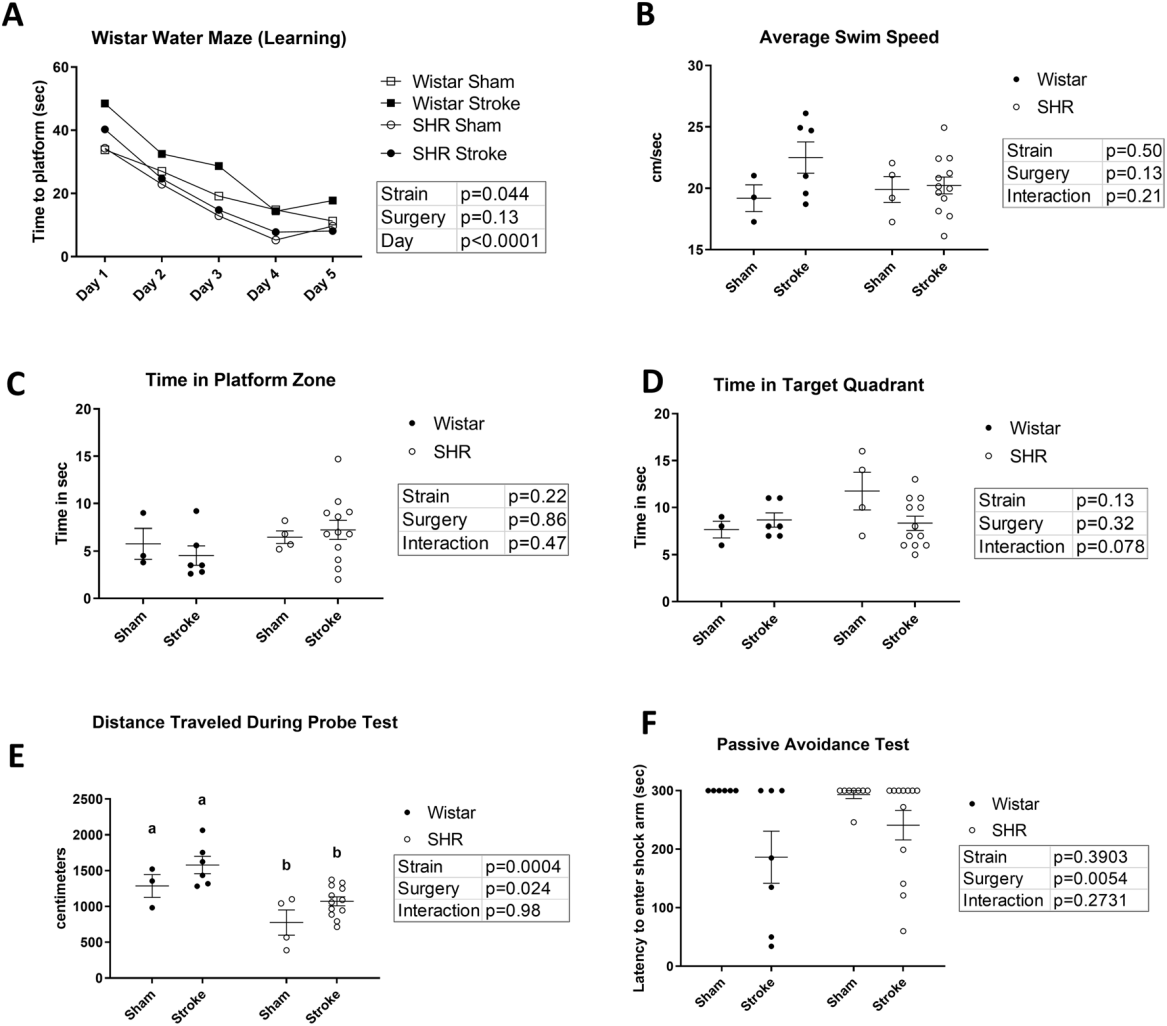
Stroke causes long-term fear associated memory dysfunction and hypertension induces behavioral despair. (**A**) Time to reach the platform during the acquisition of the Morris Water Maze was recorded. All groups showed a consistent learning curve (P for time < 0.001, 2-way ANOVA). (**B**) Average swim speed over the course of the experiment was measured for all groups. (P > 0.05, 2-way ANOVA) (n = 6–12 per group). (**C**) Time spent in the platform zone and (**D**) time spent in the target quadrant were measured in the probe trial to examine the memory function (P > 0.05, 2-Way ANOVA). (**E**) Total distance travelled during the probe test was a measurement for behavioral despair and depression. (P for strain < 0.001, 2-way ANOVA). (a,b: Tukey post-hoc multiple comparisons, pairs of means with different letters are significantly different) (**F**) Passive Avoidance showed a decrease in the average latency to enter the shock arm for stroked animals. (P < 0.01, 2-way ANOVA) (n = 6–12 per group).

To assess the spatial learning and memory, animals were tested using the Morris Water Maze at week 4. All groups showed a significant learning behavior (P < 0.0001) (Fig. 3A) and similar average swim speeds over the course of the experiment (Fig. 3B). There was no significant difference between the four groups during the probe-trial in the time spent in the platform zone or the time spent in the target quadrant, indicating an intact memory function. (Fig. 3C,D) Despite this, SHR animals exhibited a significant reduction in the total distance traveled during the probe test, regardless of the surgery, indicating a significantly impaired exploratory behavior, a sign of “behavioral despair” and depression^33^ (Fig. 3E).

### Stroke induces long-term memory impairment in both hypertensive and normotensive animals

Long-term memory was assessed using the passive avoidance test starting at week 4 post-stroke. Both strains of stroked animals showed a significant reduction in their latency to enter the shock arm, 3 days after receiving the shock, indicating an impairment in their avoidance-driven long-term memory (P = 0.0054). Animals that entered the shock arm immediately after being introduced into the arena (< 30 s latency) had their trials excluded from the results of this study (1 animal) (Fig. 3F). Since this test has a pronounced learning effect, it was not repeated.

### Stroke accelerates grey matter atrophy in hypertensive animals

**Figure 4:**
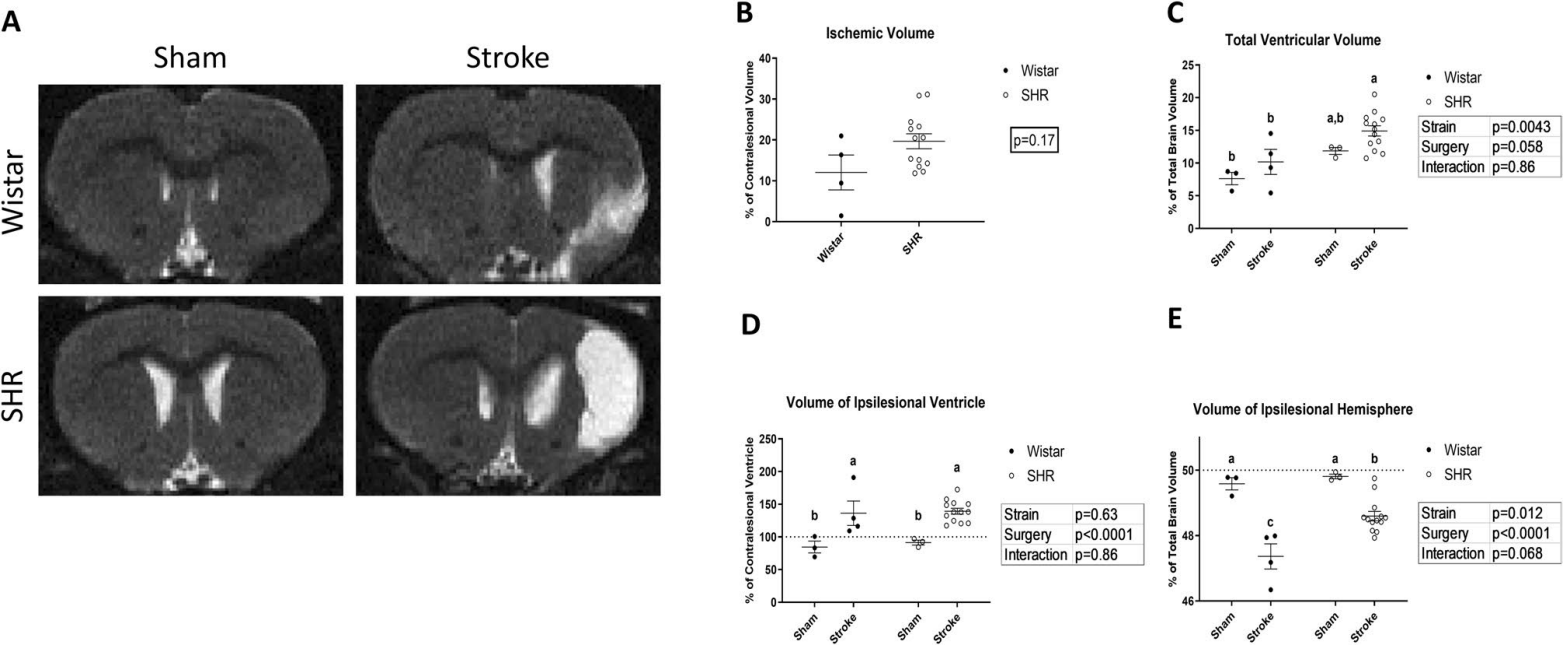
Stroke induces enlarged ventricles and grey matter atrophy in SHRs, as measured by MRI at 12-weeks post-stroke. (**A**) Representative images for diffusion weighted MRI, week 12 post-stroke. White indicates areas with high water content. (**B**) Ischemic tissue as % of the volume of the contralesional hemisphere (P > 0.05, t-test) (n = 6–12 per group). (b) Total volume of lateral ventricles as % of total brain volume, with SHR showing significant bilateral enlargement of lateral ventricles (P for strain < 0.01, 2-way ANOVA). (**D**) Volume of the ipsilesional ventricle as % of the contralesional ventricle, with significant enlargement for stroked animals in both strains. (P for surgery < 0.001, 2-way ANOVA). (**E**) Total volume of the ipsilesional hemisphere as % of total brain volume, decreased for stroked animals in both strains. (P for surgery < 0.001, 2-way ANOVA). (a,b,c: Tukey post-hoc multiple comparisons, pairs of means with different letters are significantly different).

The animals were assessed via T2-weighted MRI on the brain at 12 weeks post-stroke to assess brain morphological changes (Fig. 4A). Due to small numbers in the Wistar group, the larger ischemic infarct size in the SHR animals did not achieve statistical significance (Fig. 4B). The total volume of lateral ventricles, an indicator of cerebral atrophy, was significantly larger in SHR animals compared to Wistars, and this was more prominent in the stroked animals (Fig. 4C). Interestingly, this difference was not apparent when comparing the size of the ipsilesional ventricles alone (Fig. 4D), where both strains showed a marked enlargement over their sham counterparts. This indicates that the loss of tissue in SHR animals is not confined to the stroked hemisphere, but also affects the contralesional hemisphere in a significant way. As the total ventricular volume quantified includes the two lateral ventricles, as well as the third central ventricle; and by subtracting the volume of the ipsilateral lateral ventricle from the total ventricular space, we can see that the significant difference found in Fig. 4C was due to the difference in the size of the contralateral ventricle and the central third ventricle, indicating a generalized atrophy effect for stroke in hypertensive animals. In both strains, as expected, the ipsilesional hemisphere was significantly atrophied compared to the contralesional one (Fig. 4E).

We performed an MRI scan on all the excluded animals and found that only 1 out of the 12 excluded animals showed signs of a very minor ischemic damage in the brain, which added to our confidence in the validity of our exclusion criteria. (data not shown).

### Hypertension contributed to heightened apoptosis in the ischemic borderzone at 12 weeks following stroke

**Figure 5:**
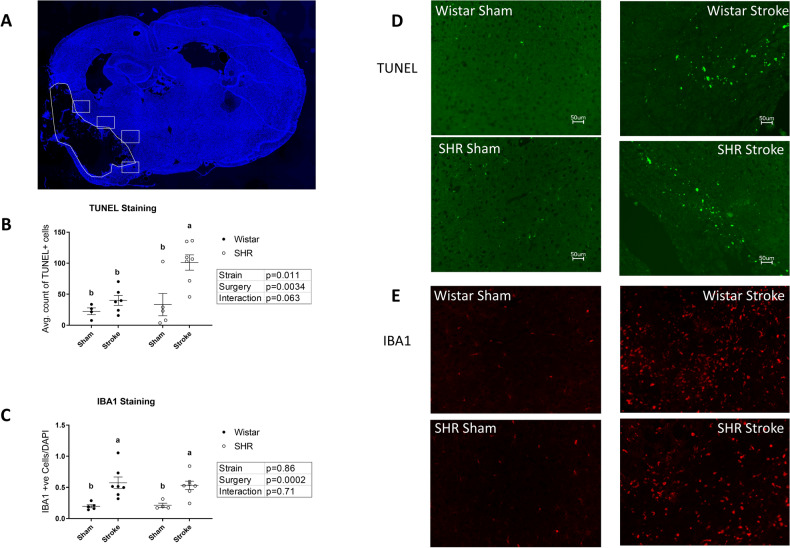
Stroke induces neuronal apoptosis in the ischemic border zone region of brain tissue, which is exacerbated by hypertension. (**A**) Representative image of the positions from which the TUNEL and IBA1 images were taken. The polygon outline represents the ischemic tissue, the squares represent fields for quantitation. (DAPI) (**B**) SHRs displayed a significant increase in apoptosis (P < 0.05, 2-way ANOVA) compared to normotensive animals. Stroked animals displayed a significant increase in apoptosis (P < 0.01, 2-way ANOVA) compared to Sham animals. (n = 4–8 per group). (**C**) Stroked animals displayed a significant increase in IBA1 staining compared to shams (P < 0.001, 2-way ANOVA) (n = 4–8 per group). (a,b: Tukey post-hoc multiple comparisons, pairs of means with different letters are significantly different.) (**D**) Representative images from TUNEL staining. (**E**) Representative images from IBA1 staining (false color).

Quantification of TUNEL staining in the ischemic borderzone (Fig. 5A), showed increased apoptosis in SHR animals compared to Wistars (Fig. 5D), and both stroke groups exhibited a marked increase in apoptosis compared to their sham counterparts. Stroke exacerbated the effect of hypertension significantly when comparing the stroked SHRs to the stroked Wistars (Fig. 5B).

IBA1 is a marker of microglia/macrophage activation. To assess whether the increase in apoptosis was associated with increased inflammation, IBA1 positive cells from the ischemic borderzone (Fig. 5A) were quantified (Fig. 5E). Even 12 weeks after stroke, there was ongoing inflammation, IBA1 positive cells, in both hypertensive and normotensive animals to a similar extent (Fig. 5C). No significant difference was found in the levels of the pro-inflammatory cytokines IL1B and TNFa between groups using ELISA on the whole-brain homogenate of the ipsilesional brain hemisphere. (Supp. Fig. 3).

### Hypertension increases the late expression of markers of DNA damage and cell death after stroke, which are not increased in normotensive animals

**Figure 6:**
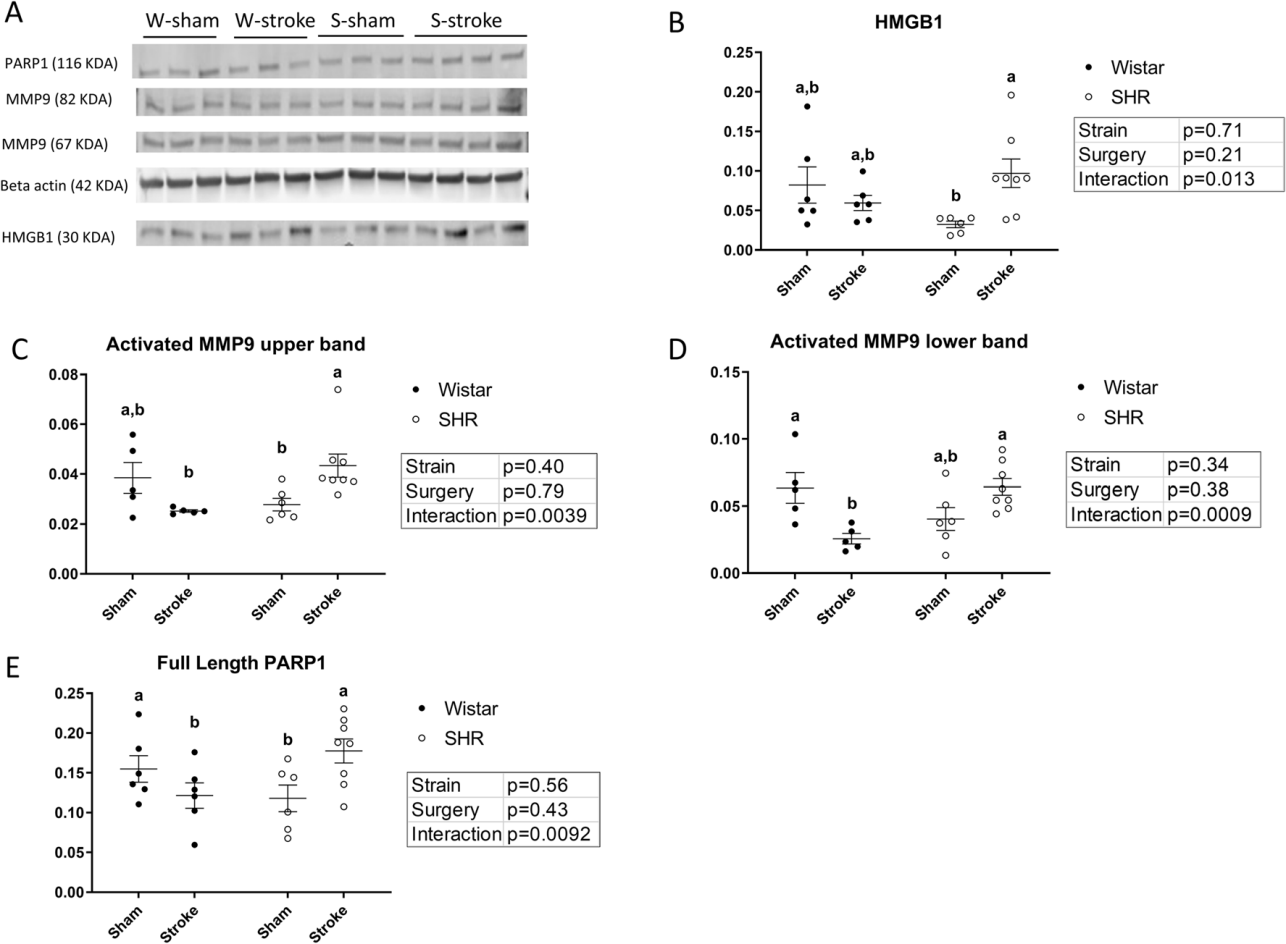
SHRs display increases in markers of neuronal damage following stroke that are not increased in normotensive animals. (**A**) Representative image for Western blot data. (**B**) Stroked SHR show increase in HMGB1 (p (interaction) < 0.05, 2-way ANOVA). (n = 4–8, per group). (**C**) and (**D**) Stroked SHRs show increase in activated MMP9 (p (interaction) < 0.01 for upper band and < 0.001 for lower band, 2-way ANOVA). Stroked SHRs show an increase of activated MMP9 (p < 0.05 for upper band and < 0.01 for lower band, Bonferroni post-hoc test) (n = 4–8, per group). (**E**) SHR show increase in full-length PARP1 after stroke (p (interaction) < 0.01, 2-way ANOVA). Stroked SHR show an increase of PARP1 compared to stroked Wistars (p < 0.05, Bonferroni post-hoc test) (n = 4–8, per group). (a,b: Tukey post-hoc multiple comparisons, pairs of means with different letters are significantly different).

Pharmacological poly(ADP-ribose)polymerase-1 (PARP1) is a marker of DNA damage that is associated with increased inflammation and the accumulation of reactive oxygen species^28–30^. We found that stroke caused a significant increase in the expression of PARP1 in the brain homogenate of SHRs, an effect that was not present in normotensive animals (Fig. 6A). There was no difference in the expression level of cleaved (inactive) PARP1 (data not shown).

In SHRs, increased High mobility group box 1 protein (HMGB1) expression has been shown to be associated with neuronal damage, increased cell death and increased inflammation^31^. We found that stroke significantly increased the expression of HMGB1 in the brain homogenates of SHRs compared to shams, but not in normotensive animals (Fig. 6B).

Matrix metalloproteinase 9 (MMP-9) is a member of the zinc-metalloproteinases family involved in the degradation of the extracellular matrix. In this study, hypertension was associated with increased expression of the cleaved active forms of MMP9 at 12 weeks after stroke, but this was not the case in normotensive animals. Furthermore, stroked SHRs displayed a significant increase in the expression of activated MMP9 compared to their sham counterparts, showing that this was not due to hypertension alone. (Fig. 6C,D).

## Discussion

Post-stroke cognitive and psychological problems are one of the most prevalent yet understudied aspects of stroke-related disability in human patients. In order to be able to develop treatments for PSCI, it is important to develop reliable animal models suitable for translational studies. Although numerous studies investigated the effects of stroke and MCAO on the development of post-stroke cognitive impairment^32,33^, most studies used relatively healthy animals and concluded by 30 days post-stroke. In humans, most patients suffer from comorbid diseases like hypertension and diabetes and although maximum motor recovery is usually achieved within 6 months to one year after stroke, stroke survivors display increased incidence of cognitive impairment and dementia for decades after the initial event^34^. We now know that this is likely due to long term progressive neurodegeneration, which is exacerbated by comorbidities^12^. The deleterious effect of hypertension on the development of cognitive impairment, even in the absence of stroke is well established^18^. However, it is usually studied in the context of assessing the effect of blood-pressure lowering medications on preventing these deleterious effects. Our study is one of the very few studies to compare normotensive and SHRs, with and without stroke, in order to determine the extent of contribution of hypertension and stroke to the development of cognitive impairment. Hypertension is known to worsen stroke outcome and increase mortality acutely following MCAO^35,36^. In this study, we started by measuring the mean arterial blood pressure of a separate cohort of animals, of the same weight and age to our main SHR cohort, using telemetry, to prove that the animals had already developed hypertension before the initiation of the MCAO. This was done because the stress generated by telemetry was expected to impact the behavioral data. Although our lab has extensive experience working on both normotensive and hypertensive animals, comparing Wistar and SHR animals directly in a head-to-head fashion presented a unique challenge. Although a 90-min MCAO was found to reliably produce cognitive deficits in Wistar rats, it resulted in a high rate of mortality in SHRs, and while 60-min MCAO produced very little mortality in SHRs, Wistars had a high rate of spontaneous, 24-h recovery. We elected to use the 60-min MCAO to reduce animal mortality, excluding spontaneously recovering Wistar animals from further analysis. Wistar rats were selected for this comparison instead of Wistar Kyoto, as Wistar Kyoto animals are known to have intrinsic problems in neurobehavior, regardless of hypertension^37^.

The choice of our timepoints for cognitive assessment was made taking into consideration the suitability of those behavioral tests for repetition, as well as the effect of the different tests on each other. For example, with more repetition we found that animals lost interest in exploring the objects in the NOR, which prompted us to retire the test from week 3 to week 12. Even with this long break, overall object exploration was decreased for all animals at week 12. Similarly, we found that the PAT and the MWM produced a great physical and psychological stress for the animals, thus potentially affecting other behavioral tests that could have been done in the same time period. Therefore, no other tests were administered concurrently with the PAT and MWM.

Our main hypothesis was that hypertension would exacerbate the deleterious effects of MCAO on memory and learning, based on our previously published findings in SHRs^21,25^. Both SHRs and Wistars in this study developed impairments in learning and memory at 4 weeks after stroke, as demonstrated in the passive avoidance task, but the binary outcome (yes/no) was not conducive to detecting differences between groups with impairment. We were unable to demonstrate deficits due to stroke in NOR task in either strain, however, when tested at 3 and 12 weeks after stroke. Our previous investigation revealed profound deficits in NOR at 30 days in a similarly young SHR cohort^21^. Whether our failure to replicate that finding was due to the differences in timing of the testing (3 and 12 weeks vs. 30 days) or the experimental set-up remains unclear. It is also possible that depression-like behavior animals develop has a big impact on NOR and water maze tests which limits our ability to detect cognitive deficits. Additional tests designed to evaluate the depression-like behavior need to be incorporated into future studies that monitor long term outcomes. While our previous study did not include a Wistar comparison group^21^, in the current study the PAT findings remained robust, assuring us that both strains developed PSCI. It is worth noting that our animals were housed individually following MCAO due to concerns that housing the animals in pairs would be dangerous due to the wounds present, and concerns regarding post-stroke feeding competition and recovery. We consider this to be one of the limitations of our study as social isolation is known to be an influencing factor on animal behavior in cognitive tests^38^. However, the differences in behavior we found between the experimental groups were all among animals of the same housing conditions, leading to our conclusion that the behavioral differences observed were a result of strain differences rather than the housing conditions.

This study is one of the first studies to establish the ongoing deleterious long-term effects of hypertension on recovery of the stroked brain. Although others have reported changes over time after stroke, they failed to include sham groups, so the effects of hypertension alone could not be teased out^39,40^. It is known that SHR animals develop enlarged brain ventricles over time^41^, however in our study, the ventricular volume was not significantly different between the Wistar and SHR sham groups, which points to the fact that our SHR animals have not yet spontaneously developed larger ventricles at the time of the study. Similarly, the increase in the ventricular size in the ipsilateral hemisphere was similar in both strains. We found that SHRs had a significant increase in the total ventricular size of both hemispheres, which did not disappear after standardizing on the ventricle size of the strain using the sham animals. We found that SHR animals had profound brain atrophy, in both hemispheres, at 12-weeks post-stroke, when compared to normotensive animals and even to sham-operated SHRs. This indicates that hypertension prolongs the ongoing neurogenic damage of stroke, and this continues to the late time-points. Our findings point to SHR animals being uniquely more vulnerable to the stroke induced ventricular enlargement, specifically in the contralateral hemisphere, which can possibly be attributed to the increased apoptosis post stroke. Our original hypothesis was that this damage was immunologic in nature due to increased inflammation in SHRs, however, our results obtained 12 weeks post-stroke indicate similar levels of post-stroke inflammation. Flow cytometry may have been able to better describe the nature of the activated microglia and macrophages in our samples, but we did not collect the tissue in a way that would allow that analysis. In our previous investigations, we showed that PSCI was associated with ongoing microglial activation and cell death at 30 days in SHRs, and it could be therapeutically targeted^21^. Here we see that a long-term increase in apoptotic cell death is more pronounced in SHRs. It was ideal to perform detailed histological analyses on the hippocampus to determine the extent of hippocampal damage. However, in most of our animals, the ischemic lesion was big enough to reach sub-cortical areas including destroying all or parts of the ipsilateral hippocampus, which made quantifiable analyses of hippocampal injury impossible for these animals.

**Figure 7:**
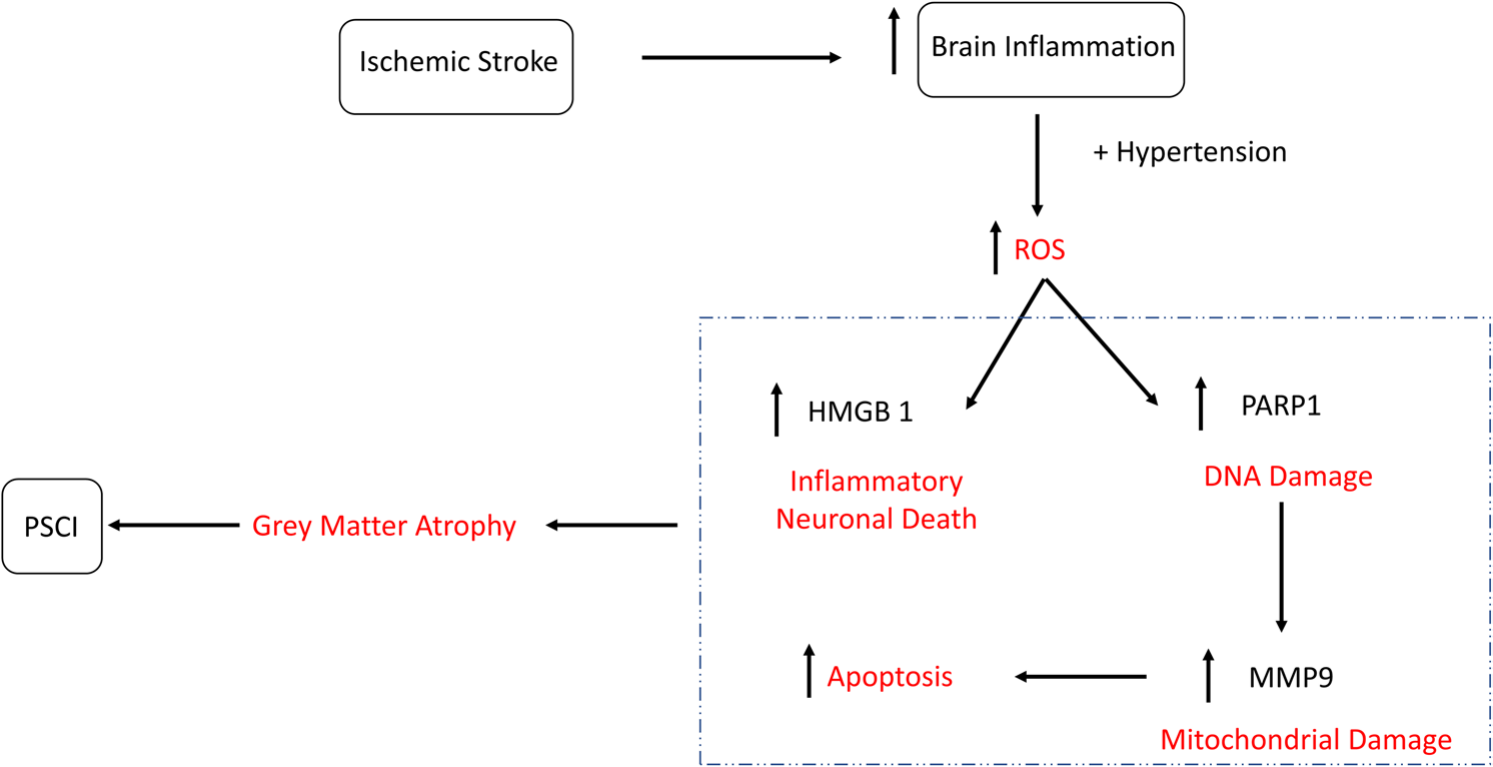
Stroke induces long term neurodegeneration in hypertensive animals. Ischemic stroke causes a marked inflammation in stroked animals. In hypertensive animals, which already suffer from an increase in ROS production, the ischemic insult and the oxidative stress result in chronic increase in DNA damage and neuronal cell death, marked with an increase in PARP1 and HMGB1. PARP1 increase induces the transcription of MMP9, which triggers apoptosis. The result of these processes is chronic neuronal loss in the form of grey matter atrophy.

The increase in PARP1, MMP9 and HMGB1 associated with stroke in SHRs points to the unique relationship between hypertension and the enhancement of post-stroke cell death (Fig. 7). In addition, PARP1 plays a role in the development of a caspase-independent form of cell death, termed as parthanatos, in response to ischemia/reperfusion damage^42,43^. Chronic inhibition of full-length (active) PARP1 in SHRs was found to reduce hypertension-related tissue damage in the brain and vascular tissue without affecting the blood pressure^44^. Both PARP1 knock-out and PARP1 inhibition are known to provide anti-inflammatory and neuroprotective effects in models of traumatic brain injury^45^ and cerebral ischemia^46^.

In addition to its well-known deleterious effects on acute ischemic stroke, including disruption of the blood brain barrier (BBB), increased risk of hemorrhagic complications, and worsened stroke outcome^47,48^, MMP9 expression has been found to be increased in SHR brains after MCAO, and associated with increased deficits in memory and learning^49^. In addition, it plays an important role in the cleavage of PARP1^50^. Although the increase in MMP-9 after acute ischemia has been well documented, the increased activity has been reported as transient, returning to baseline in the week following stroke^51,52^. However, MMP9 expression is increased in several chronic inflammatory CNS pathologies such as multiple sclerosis and Devic's neuromyelitis optica^42^. It has been reported that PARP1 inhibitors also inhibit MMPs, indicating that the beneficial effects of PARP1 inhibitors after stroke may be in part due to the inhibition of MMPs^53^. MMP9 is secreted as an inactive pro form, which is cleaved and activated, appearing as two bands of cleaved MMP9^54^. MMP9 was found to play a critical role in the development of age-dependent post-operative cognitive decline, and MMP9 knockout mice were found to be protected from this phenomenon^55^. Lastly, MMP9 plays an important role in the secretion of pro-inflammatory cytokines and can act as one itself, particularly in response to tissue injury^56^.

HMGB1 is a protein involved in DNA organization and transcription regulation in the nucleus^57^. In the brain, HMGB1 acts on microglia to mediate chronic neuroinflammation that drives progressive neurodegeneration^58^. HMGB1 was also found to be elevated in models of traumatic brain injury, neuroinflammation, epilepsy, and cognitive dysfunction^59,60^. Finally, HMGB1 was found to be released from necrotic cells in the ischemic core, activating an early inflammatory response and its concentrations were found to correlate with disease severity and outcome after brain injury^61^.

Our study demonstrated that comorbid hypertension not only worsens the initial injury due to stroke, it exacerbates ongoing tissue damage that occurs months after motor recovery. Although cognitive impairment was evident in both Wistars and SHRs at 30 days after stroke, molecular evidence of active neurodegeneration was more than twofold higher in the SHRs at 12 weeks. In fact, the statistically significant “interaction” we report suggests that the presence of hypertension actually reverses the normal response of these mediators to ischemia and reperfusion. While we found significant histological differences between the normotensive and hypertensive animals, these histological findings did not directly correlate to a difference in cognitive impairment, probably due to the relative insensitivity of the testing methods. It is still likely that hypertension contributes to progressive post-stroke cognitive impairment and differences may have been demonstrated with more sensitive tests or longer-term follow-up.

## Perspectives

Our study aimed to establish the contribution of hypertension to the clinically relevant phenomenon of delayed PSCI. We found that delayed cognitive impairment develops in both normotensive and SHRs. However, SHRs showed an increase in delayed DNA damage and cell death, resulting in overall tissue atrophy which was greater than that seen in hypertension alone. Neurodegeneration is a LATE target for intervention after stroke in hypertensive individuals.

## Supplementary information

Supplementary Information
