# Supplementary material for: Stroke promotes the development of brain atrophy and delayed cell death in hypertensive rats
Source: Sci Rep. 2020 Nov 19;10:20233. doi: 10.1038/s41598-020-75450-6 (PMC7678843; doi:10.1038/s41598-020-75450-6)

Stroke promotes the development of brain atrophy and delayed cell death in hypertensive rats: relevance to cognitive and psychological outcomes

Mohammed A. Sayed[1,2], Wael Eldahshan[1,2], Mahmoud Abdelbary[3], Bindu Pillai[1,2], Waleed Althomali[1,2], Maribeth H. Johnson[4], Ali S. Arbab[5], Adviye Ergul[6,7], and Susan C. Fagan* [1,2]

[1]Clinical and Experimental Therapeutics, College of Pharmacy, University of Georgia, Augusta, GA; [2]Charlie Norwood VA Medical Center, Augusta, GA; [3]Department of Physiology, Medical College of Georgia, Augusta, GA; [4]Department of Neuroscience and Regenerative Medicine, Augusta, GA; [5]Georgia Cancer Center, Augusta, GA; [6]Department of Pathology and Laboratory Medicine, Medical University of South Carolina, Charleston, SC; and [7]Ralph H. Johnson VA Medical Center, Charleston, SC

Corresponding Author: **Susan C. Fagan, Pharm.D., FAHA**

914 New Baillie Street, HM Building Room 116, Augusta, GA 30901

Tel.: 706-721-0130 Email: sfagan@uga.edu

Short Title: Hypertension and Post-Stroke Cognitive Impairment

Manuscript Word Count: 5998

Total Number of Figures: 7

Supplementary Figure 1

A

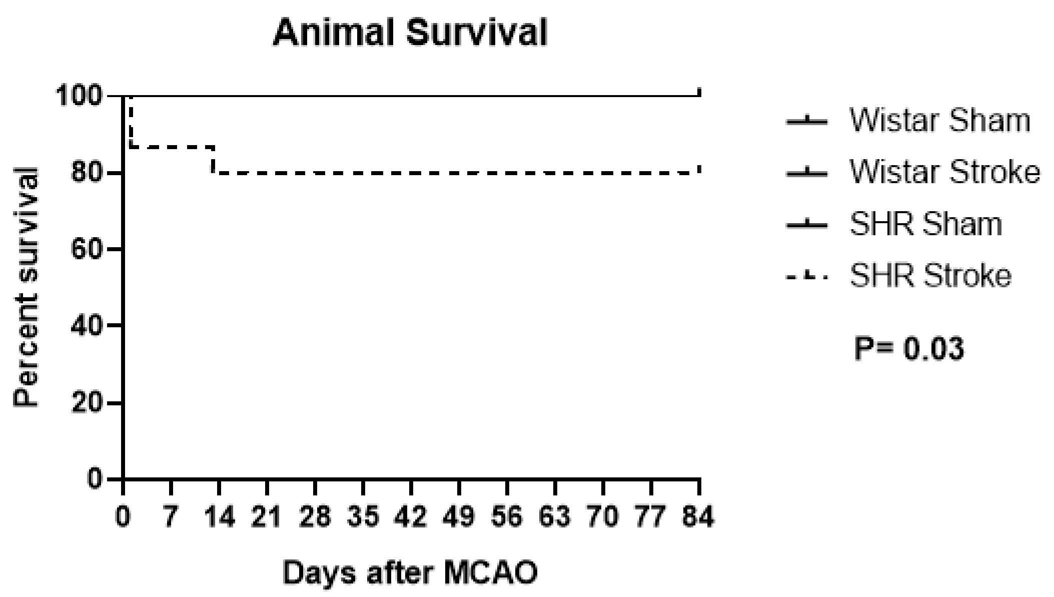

Supplementary Figure 2

A

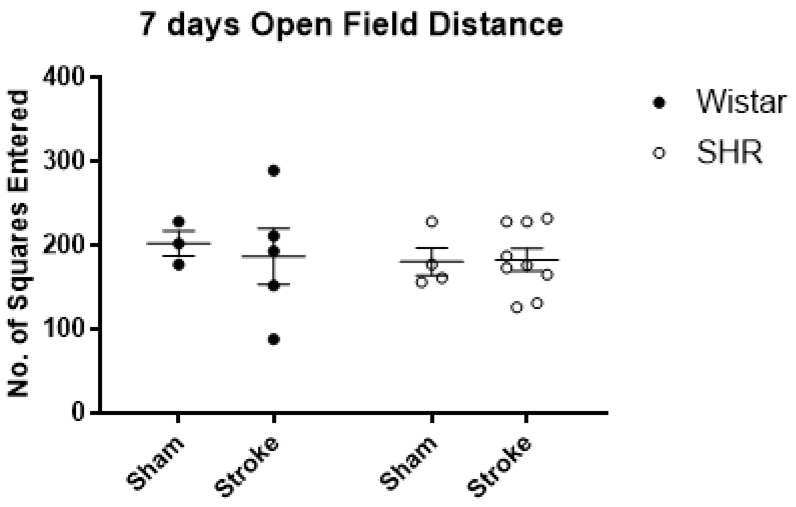

Supplementary Figure 3

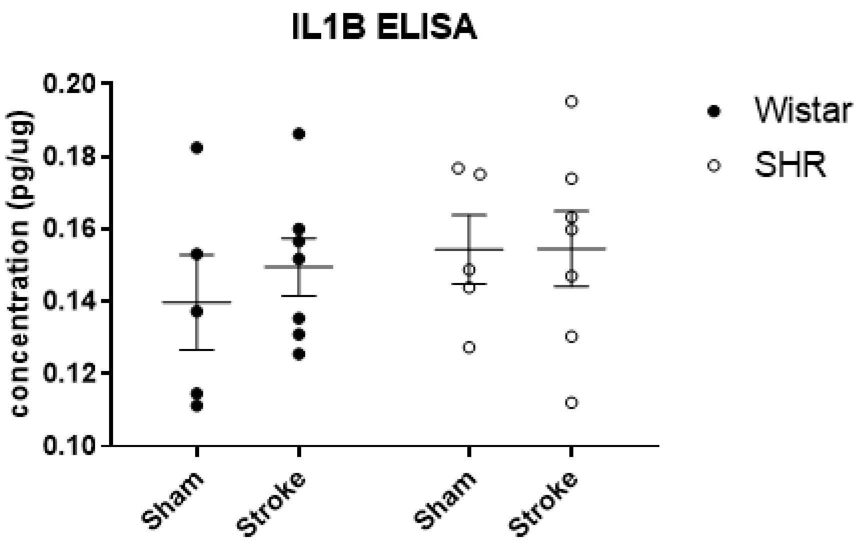

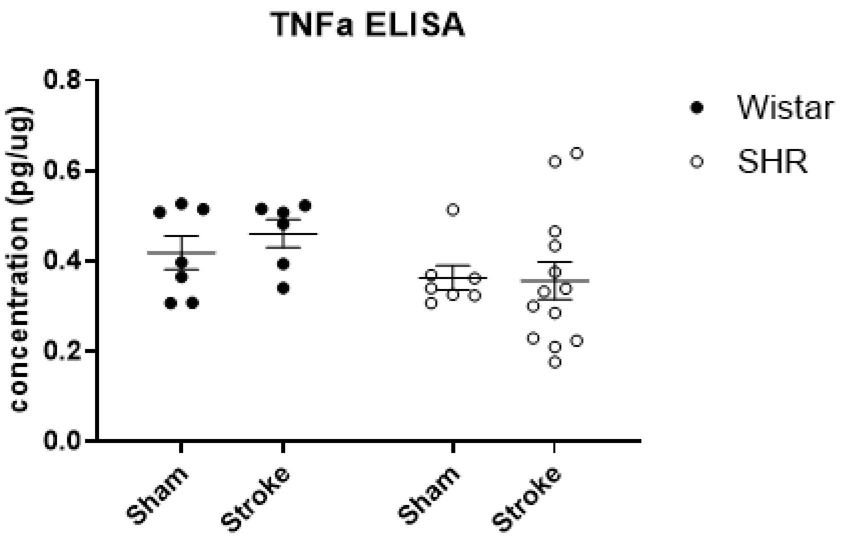

Supplementary Figure 4

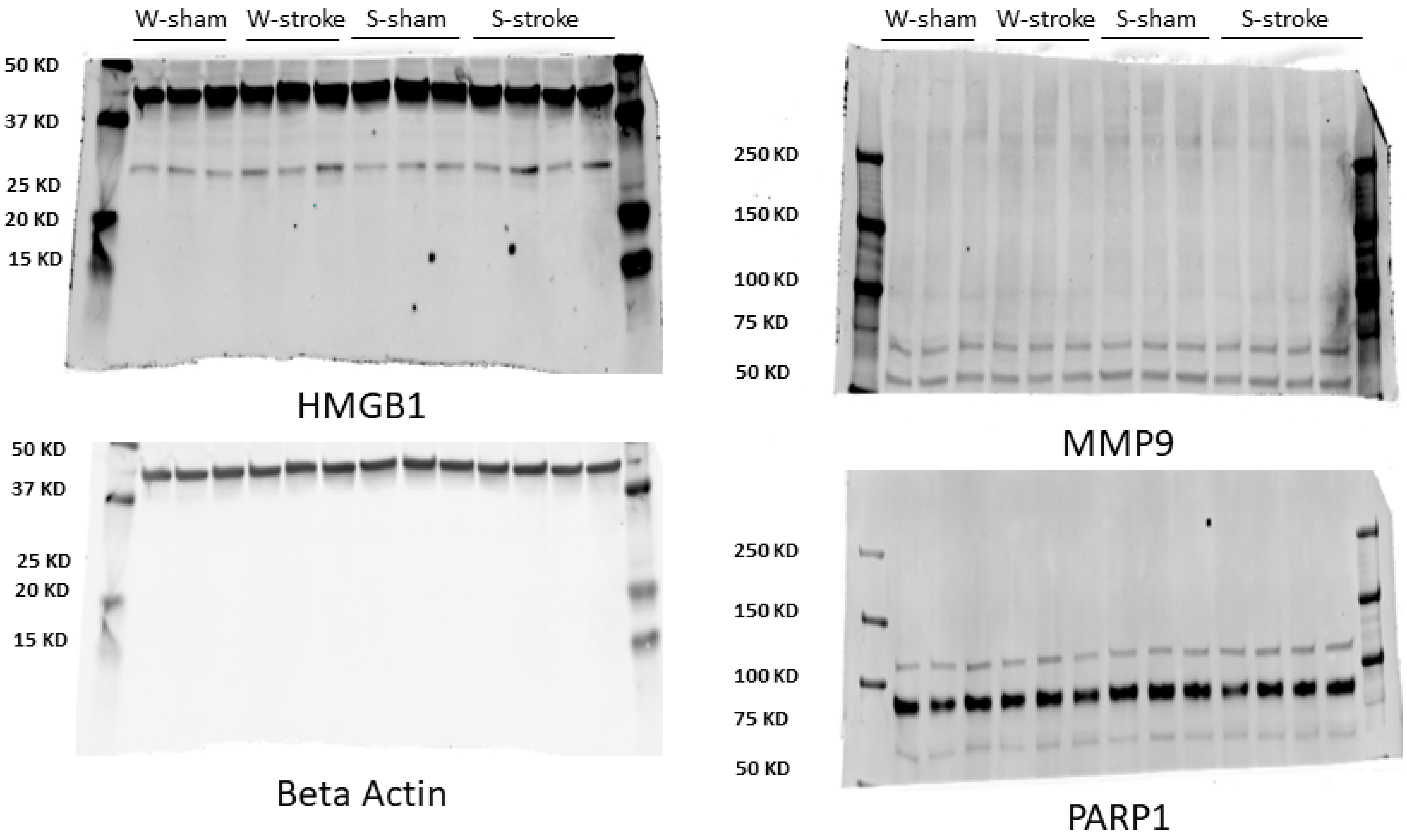

Supplement: Supplementary file 1 — Supplementary Information [file 41598_2020_75450_MOESM1_ESM.pdf]
